# Metabolomic evaluation of selenium seed priming on mitigating lead stress toxicity in *Vicia faba* plants

**DOI:** 10.1186/s12870-025-06453-6

**Published:** 2025-04-17

**Authors:** Asmaa Abdelsalam, Arezue Boroujerdi, Elham R. S. Soliman

**Affiliations:** 1 Botany and Microbiology Department, Faculty of Science, Helwan University, Helwan, 11795 Egypt; 2 Chemistry Department, Claflin University, Orangeburg, SC 29115 USA

**Keywords:** Amino acids, Epinephrine, Heavy metals stress, Metabolic pathways, Metabolites, NMR spectroscopy, PCA

## Abstract

**Background:**

Persistent lead contamination and the absence of natural remediation elements exacerbate the long-term toxicity of plants. Nevertheless, it has been consistently shown that selenium has a protective effect against heavy metal toxicity in plants. Consequently, it is imperative to identify the metabolic pathways that selenium employs to enhance the resistance of plants to lead stress. This study aimed to investigate the metabolomic alterations induced by selenium priming of *Vicia faba* seeds to enhance their tolerance to lead stress.

**Results:**

Selenium seed priming significantly improved the growth parameter and mitigated the adverse growth consequences observed under lead stress. Nuclear magnetic resonance-based metabolomic analysis identified 58 metabolites in the polar extracts of the shoots, with the metabolites composed of amino acids (40%), carboxylic acids (12%), fatty acids (11%), carbohydrates (5%), alkaloids (5%), and phenols (4%). The addition of Pb facilitated the biosynthesis of unique metabolites, including 2-methylglutarate, 3-methyladipate, and epinephrine, which were absent in control and selenium-treated samples. Conversely, 4-aminobutyrate and 2-methylglutarate were entirely absent in Pb samples. Selenium-treated plants accumulated trigonelline and AMP at levels 1.4 and 6.0 times, respectively, more than the control samples. Selenium-primed plants exposed to lead stress exhibited higher levels of asparagine, tryptophan, and xanthine compared to other treatments. As determined by both enrichment analysis and pathway analysis, the most significantly altered pathways were alanine, aspartate, and glutamate metabolism; aminoacyl-tRNA biosynthesis; and valine, leucine, and isoleucine biosynthesis pathways.

**Conclusion:**

The results demonstrate the crucial role of selenium priming in enhancing the growth and lead stress resistance of *Vicia faba* plants by significantly altering the concentrations of key metabolites and metabolic pathways, particularly those involved in amino acid metabolism, offering a promising strategy for improving plant resilience to heavy metal contamination.

**Supplementary Information:**

The online version contains supplementary material available at 10.1186/s12870-025-06453-6.

## Introduction

Faba bean (*Vicia faba*, Family: Fabaceae) is an important crop for human and cattle nutrition. It maintains soil fertility by increasing the quantity of nitrogen in the soil through nitrogen fixation and release from its residues after the crop has been harvested [1]. The dried seed is rich in fibers, carbohydrates, proteins, alkaloids, terpenoids, and polyphenols [2]. It accounts for 29.53% of global leguminous yield production. It has been largely produced in countries of Asia, Africa, and Europe, with China being the leading producer [2].

Lead (Pb) is a heavy metal that accumulates over time and becomes mobilized within its surroundings. Pb pollution is mostly caused by Pb-containing paint, engine emissions of volatilized leaded gasoline, mining, smelting, battery recycling, waste incineration, and Pb-based insecticides. The persistence of Pb in soil and the absence of natural remediation elements contribute to the ongoing problem associated with the previous uncontrolled use of Pb pollution sources [3, 4]. Two routes of Pb penetration in plant tissue are via the leaf and root. Foliar spray involves the large surface area of plant leaves absorbing metal ions from polluted air through their stomata and cuticular cracks. The contaminants are then translocated via the vascular system by shoot–root translocation. The root system provides another point of entry where Pb is absorbed, along with soil nutrients, and translocated to all plant tissues via root-shoot translocation. Once absorbed by the plant, Pb can bind to the endodermis, preventing the movement of Casparian strips, and bind to the cell wall and plasma membrane [5, 6]. Pb contamination inhibits ATP synthesis, lipid peroxidation, Calvin cycle enzymes, and the absorption of critical minerals such as magnesium and iron, while inducing the creation of reactive oxygen species (ROS), which cause DNA damage. It has a significantly negative effect on seed germination, plant growth and development, crop yield, transpiration, cell permeability, protein content, and stomatal closure. Additionally, it results in distortion of the chloroplast ultrastructure, which slows the photosynthesis process [7, 8]. Numerous studies have shown that Pb exposure significantly alters plant metabolic profiles. Zhang et al. [9] reported that different forms of Pb (nano-Pb, mic-Pb, and PbCl_2_) affected eggplant leaves, with mic-Pb and PbCl_2_ causing more similar metabolic changes than nano-Pb. It is intriguing that the PbCl_2_ treatment resulted in specific alterations in metabolites associated with the TCA cycle and pyrimidine metabolism. In a similar vein, Wang et al. [10] implemented GC–MS to investigate the metabolomic response of radish roots to Pb and Cd stress. Their findings suggested that carbohydrates, amino acids, and organic acids were the primary targets of heavy metal stress. Pathway analysis also demonstrated that Pb stress brought about significant modifications in the metabolism of carbohydrates, energy, and glutathione. Pidatala et al. [11] demonstrated that Pb^2+^ significantly induced important metabolic pathways, including glucose metabolism and amino acid metabolism, in vetiver leaves and roots.

Selenium (Se) is a trace element that is essential for humans and beneficial for enhancing plant growth and tolerance to stress conditions. Principally, selenium, in the form of selenate (SeO_4_^2^⁻) or selenite (SeO_3_^2^⁻) salts, has excellent solubility, stability, and potential mobility in natural environment [12]. Se-biofortification of plants has been recognized as an innovative method for generating Se-enriched agricultural products which enhanced crop nutrition quality as well as stress tolerance [13].

The application of exogenous Se to plants via foliar spray, seed priming, or soil mixing has dose-dependent beneficial and toxic effects [14]. Se concentrations in soils typically range from 0.01–2 mg/kg, with concentrations greater than 3 mg/kg considered toxic to plants. Se functions as an antioxidant in plants and confers tolerance to various abiotic stresses, including salinity [15], drought [16], heat [17], and heavy metal [18, 19] stresses. Its protective mechanism entails an increase in photosynthetic pigment synthesis, photosynthetic rate, gas exchange, osmo-protectant accumulation, and secondary metabolites.

Selenium, at appropriate concentrations, enhances plant resilience to heavy metal stress through a variety of mechanisms. It boosts antioxidant defense systems by increasing the activity of both enzymatic and non-enzymatic antioxidants, thereby mitigating oxidative damage induced by reactive oxygen species (ROS) generated following heavy metal stress [20]. Selenium also stimulates the production of hormones that facilitate root architectural restructuring, which in turn reduces metal uptake [21]. In rice, selenium modulates the expression of cadmium transporter genes, enhances cadmium sequestration in cell walls, and decreases cadmium bioavailability in the soil [22]. Additionally, selenium promotes the accumulation of osmolytes, assisting tomato plants in alleviating the adverse effects of heavy metal stress [23]. It further regulates the transport and distribution of heavy metal ions within plants [24]. Moreover, selenium forms complexes with heavy metals and stimulates the synthesis of phytochelatins, effectively reducing heavy metal accumulation in plants [24]. At low concentrations, Se has been reported to enhance the antioxidant capacity of stressed plants by promoting the production of metabolites such as tocopherols, flavonoids, and ascorbic acid. These metabolites play a crucial role in ROS scavenging and cellular detoxification, thereby mitigating oxidative stress [25].

Excess Se can be deleterious to plants, causing chlorosis in their leaves [26]. The determination of suitable doses and sources of Se is extremely important to ensure positive outcomes. Supplementing plants with Se was proven to reverse the detrimental effects of Pb, improve chlorophyll content, and activate antioxidants that scavenge ROS [27, 28]. The influence of Se on plant metabolic profiles has been confirmed by various studies. Liu et al. [29] documented that Se predominantly impacted the biosynthesis of amino acids, starch and sucrose metabolism, and phenylpropanoid biosynthesis pathways.

Therefore, the purpose of this study was to determine the effects of priming *Vicia faba* seeds with Se on reducing the negative consequences of Pb stress during the early vegetative stage. To understand the function and role of Se in Pb stress mitigation, a metabolomics analysis using NMR spectroscopy was performed. The metabolites that were substantially altered and differed among various treatments were identified. The affected metabolic pathways were surveyed using enrichment and pathway analysis based on the KEGG database.

## Materials and methods

### Plant growth and pot experiment

Seeds of *V. faba* L. cultivar coded "Giza 716" were purchased from the Crop Institute, Agricultural Research Center, Giza, Egypt, and planted after receiving permission. Seeds were surface-sterilized for 3 min in a 1.0% sodium hypochlorite solution and then rinsed thoroughly with distilled water. The seeds were soaked in either distilled water (Group I) or a solution of 0.1 mM sodium selenite (Na_2_SeO_3_) for 12 h (Group II). Sodium selenite was selected instead of sodium selenate, as it has been documented that selenite is less deleterious to plants. Selenium tolerance index (%) is greater for selenite than for selenate [30].

The concentration of sodium selenite was chosen since it had the highest germination rate (preliminary study) and was in accordance with Hussain et al. [31]. The seeds were planted in sterilized plant pots containing a mixture of sand and peat moss at a ratio of 2:1 and daily irrigated with tap water. Each group was comprised of a six-pot replica, with each pot containing four seeds. After two weeks, three replicates of each group (I and II) were foliar sprayed with 10 mL/pot of 50 mM lead nitrate on three consecutive days and labelled as Pb (for seeds primed with water and foliar sprayed with Pb) and SP (for seeds primed with Se and foliar sprayed with Pb). While the other set received 10 mL of distilled water/pot for foliar spraying and was labelled as C (for seeds primed with water) and Se (for seeds primed with Se). After four weeks of post-seed cultivation, plants were collected for morphological measurements, metabolite extraction, and NMR data collection.

### Morphological growth measurements

Four-week-old plants were dissected into shoots and roots, and their lengths were recorded. The fresh weight of the leaves, stems, and roots was quantified, and they were subsequently oven dried at 60 °C for two days to record their dry weights. The morphological results were expressed as a percentage of change in comparison to control plants using the following formula:

$$\% change in growth parameters= ( T - C)/ C X 100$$

“T" stands for "Treated" and represents the measured value of a growth parameter in a sample that has been exposed to a treatment (in this case, Pb, Se, or SP).

"C" stands for "Control" and represents the measured value of the same growth parameter, but in a sample that has not been treated (the control group).

### Metabolite extraction

Six replicates were chosen at random from each treatment group (C, Se, Pb, SP), and the first four leaves were collected and submerged in liquid nitrogen to stop all metabolic activities. The frozen leaf samples were kept at −80 ºC for 4 h and then lyophilized for 24 h using a Lyophilizer (Labcono, Kansas City, MO, USA). The lyophilized samples were then homogenized into powder. From each sample, 20 mg of homogenized dry plant material was used for metabolite extraction. The metabolites were extracted with a constant ratio of 2: 2: 1.8 methanol: chloroform: water (*V: V: V*) as described in Wu et al. and Kim et al. [32, 33]. The hydrophilic upper layer was separated from the extract and dried under vacuum at 30 °C (polar dry sample).

### NMR sample preparation and data collection

^1^H-NMR sample preparation, data collection and processing were carried out as described in Abdelsalam et al. [34]. Briefly, each polar dry sample was resuspended in 620 μL of NMR buffer (internal standard [1 mM TMSP, 3 (trimethylsilyl)- 2, 20, 3, 30-tetradeuteropropionic acid], 100 mM sodium phosphate buffer at pH 7.3 and 0.1% sodium azide, in 99.9% atom deuterium oxide; D_2_O). The 1D and 2D NMR data were collected at 700 MHz with a Bruker Avance™ III spectrometer. All data were acquired using a spectral width of 16.0 ppm and 64K points, yielding a 2.9-s collection time. 120 scans, 4 dummy scans, 3 s relaxation delay, and on-resonance pre-saturation at the residual water frequency were used to gather the first increment of the presat-noesy spectra for solvent suppression. Topspin 3.6.5's automatic pulse calculation experiment (pulsecal) was used to measure the 90° pulse widths for each sample (BrukerBioSpin, Billerica, MA). Bruker hsqcedetgpsisp 2.2 pulse sequence was used to gather 2D ^1^H-^13^C HSQC data. The ^1^H was detected with a spectral width of 11 ppm in the F2 channel, whereas the ^13^C was detected in the F1 channel with a spectral width of 180 ppm. Using the NMR data, bucket tables were constructed using Amix software with 0.5–10.0 ppm spectral region (excluding the water region of 4.75–4.85 ppm) and using 0.01 ppm bucket widths and the advanced bucketing option, which is based on individual peaks within the spectra that do not shift significantly (constant temperature and pH).

### Metabolite identification

Identification of metabolites was initially conducted by comparing the collected NMR data (^1^H spectra) with the 700 MHz Chenomx NMR Suite library (Chenomx Inc., Edmonton, Alberta, Canada). The identification was then confirmed through further analysis by comparing the collected ^1^H and ^1^H-^13^C HSQC spectral data with the data available in the literature and in databases (Human Metabolome Database (HMDB) and the Biological Magnetic Resonance Bank (BMRB)). The metabolites identified in this study were classified at the level of putative identification (Level 2), in accordance with the guidelines established by the Metabolomics Standards Initiative (MSI) [35].

### Enrichment and pathway analysis

Enrichment and pathway analysis were used to visualize the metabolic pathways significantly altered by Se and Pb treatment. KEGG-based metabolite sets were used for enrichment analysis. A pathway analysis was performed in reference to *Arachis hypogaea* (peanut) as the model organism.

### Data analyses

All morphological parameters data were subjected to analysis of variance (ANOVA) and means of six independent replicas were compared through post-hoc analysis using Fisher Pairwise Comparison (LSD) test at (*p* ≤ 0.05) using Minitab® software.

NMR data analyses (univariate analysis, chemometrics analysis, and clustering analysis) of the normalized NMR data were conducted using MetaboAnalyst 6.0 (MetaboAnalyst 6.0—a comprehensive server for metabolomic data analysis, (https://www.metaboanalyst.ca/home.xhtml)) [36, 37]. Principal component analysis (PCA) was adjusted to 95% confidence intervals, the statistical significances of the group patterns were evaluated using PERMANOVA and the distributions were computed using the Euclidean distance. The construction of hierarchical cluster analysis (HCA) was carried out utilizing Euclidean distance measurement and Ward clustering algorithms. Boxplots were created to identify the relative concentrations of the most significant metabolites based on their *p*-values, which were calculated using the one-way analysis of variance (ANOVA) and a post-hoc analysis which was adjusted to a significance level of p ≤ 0.05.

## Results and discussion

### Morphological changes

**Fig. 1:**
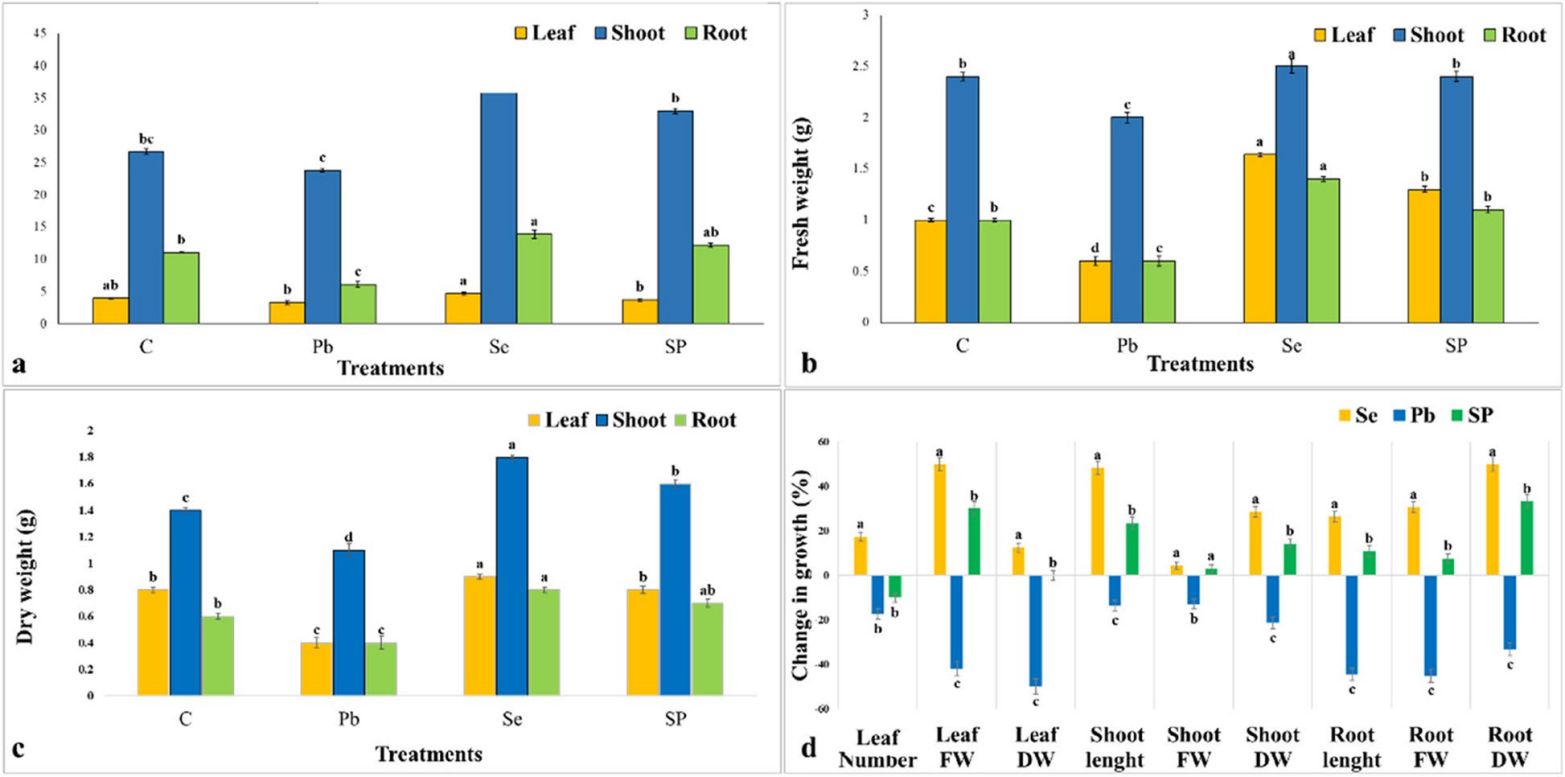
Effect of Se seed priming (Se), Pb foliar spray (Pb) and dual treatment (SP) on *Vicia faba* vegetative growth attributes compared to Control (C). **a** leaf number/plant, shoot and root length. **b** leaf, shoot and root fresh weight. **c**, leaf, shoot and root dry weight. **d**, % of changes in the measured growth parameters in different treatments (Se, Pb and SP) relative to control (C). Data are the mean of 6 replicates ± SE (standard error). The small letters (a, b, c,…) assigned to each column indicate the significance between mean of the groups being compared at *p* ≤ 0.05 level according to One-Way ANOVA

Pb samples significantly reduced the measured growth parameters to 6.1 ± 0.4 cm root length, 0.6 ± 0.04 g, 2.0 ± 0.05 g and 0.6 ± 0.05 g, leaf, shoot and root fresh weight respectively compared to 11.03 ± 0.1 cm root length, 1 ± 0.02 g, 2.4 ± 0.04 g and 1 ± 0.02 g leaf, shoot and root fresh weight respectively of the control (C) (Fig. 1 a, b). Pb stress negatively affects photosynthetic pigments and increases the production of ROS accompanied by oxidative stress damage and induces genotoxicity and DNA damage in *Vicia faba* [38]. Thus, it reduces plant growth and development [7, 8]. In contrast Se-primed seeds had a significantly positive effect on seedling growth in which, Se-treatment reached a shoot length of 39.6 ± 0.2 cm, compared to 26.7 ± 0.7 cm in control plants (Fig. 1a). Se-samples exhibited a root length of 13.9 ± 0.6 cm, versus 11.03 ± 0.1 cm in control plants (Fig. 1a). Similar response patterns were confirmed by measuring the dry weights of the leaves, shoots, and roots (Fig. 1c). Additionally, Se-primed plants show alleviating Pb stress response on the measured vegetative parameters (Fig. 1 a, b, c). The growth parameter percentage was enhanced in SP plants compared to Pb stressed plant. Pb caused 42 and 50% reduction in the fresh and dry weight of leaves while the earlier seed priming with Se (SP) significantly enhanced those to 30 and 12.5% when exposed to Pb compared to controls (Fig. 1d). These findings imply that Se priming reduces Pb-induced growth retardation, possibly through a variety of physiological and biochemical pathways that it influences within the cell [4, 39, 40]. Selenium, at low concentration, promotes plant growth by improving several physiological and biochemical processes. It enhances photosynthesis by improving chlorophyll content under heavy metal stress. Se prevents oxidative stress by improving enzymatic and non-enzymatic antioxidants, which leads to improved cell membrane integrity, reduced lipid peroxidation, and overall better growth and development of plants [41]. Additionally, Se has been shown to improve nutrient uptake and enhance the synthesis of amino acids, proteins, and secondary metabolites [25]. The application of Se resulted in notable elevation in the respiration rate in both the leaves and flowers of *Brassica rapa* plants [42]. It also improved the bean yield and delayed leaf senescence [39], enhanced root viability [40], and increased plant height and number of branches [43]. Similarly, the ameliorated effect of Se supplementation on Pb-stressed broad bean plants resulted in increased photosynthetic pigment content, enhanced CAT, GPOX, and GSH-Px activities and T-SH levels, as well as lowered concentration of ROS such as H_2_O_2_ and O_2_^•−^ as documented by Mroczek-Zdyrska et al. [44]. Several studies have reported the protective effect of Se against heavy metals e.g., Cd [45] and Cr [46].

### Metabolic changes

**Fig. 2:**
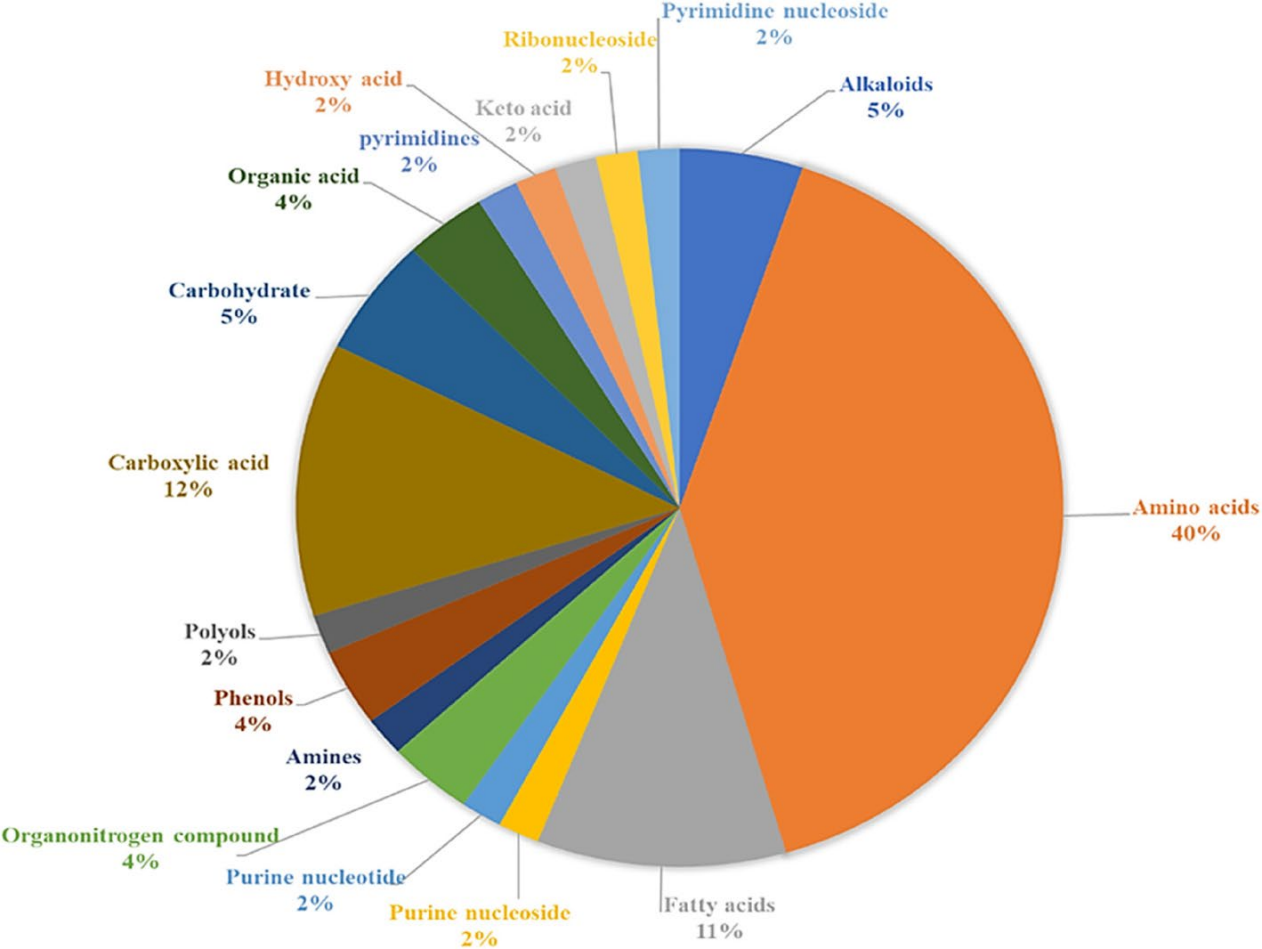
Pie chart showing the percentage of chemical classes identified in the polar extract of *Vicia faba* shoots of plants that were Se seed primed and/or Pb foliar sprayed (Se, Pb and SP) compared to controls

**Fig. 3:**
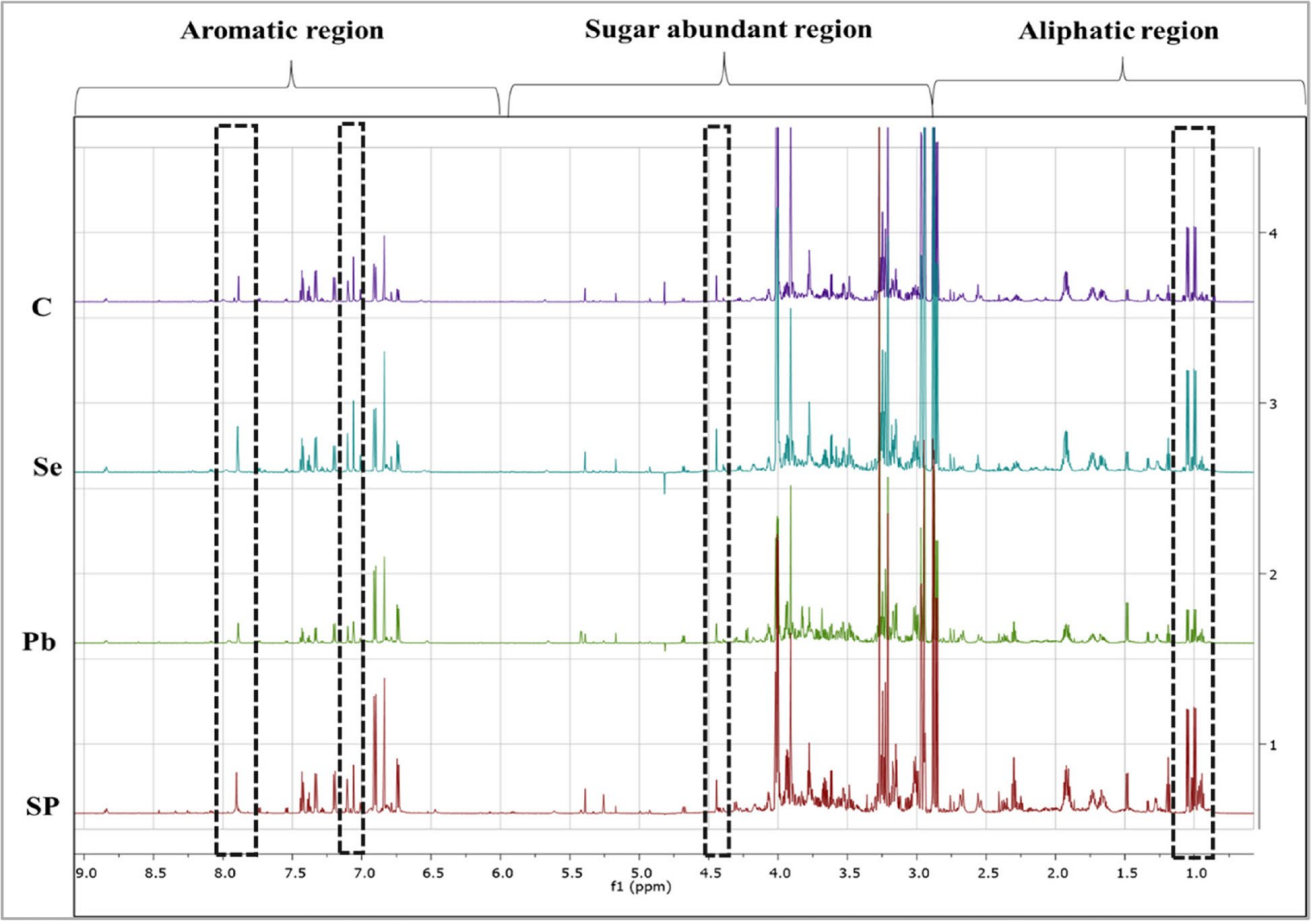
1D NMR Stacked spectra for control, Se, Pb and SP treated samples showing the metabolites variation between different groups

Untargeted metabolic profiling of the polar extract of *Vicia faba* shoots primed with Se and/or stressed with Pb was performed using 1D and 2D NMR spectra. The data generated were compared to NMR databases and literature. A total of fifty-eight metabolites have been successfully identified for all samples (treated and untreated) (Table 1). Identifying the metabolites induced during plant responses to metal stress and recovery offers insights into mechanisms that enhance plant adaptation and resistance, ultimately improving plant growth and yield. Metabolomic profiling reveals the biochemical alterations occurring within plant cells in response to various environmental factors or stimuli [47]. The metabolic profiling of faba bean shoots showed 17 different metabolic categories with the following percentages: amino acids (40%), carboxylic acids (12%), fatty acids (11%), carbohydrates (5%), alkaloids (5%), and phenols (4%) (Fig. 2). The signals identified in the spectral region of *δ* 0.5–3.0 ppm belong to aliphatic amino acids, fatty acids and amines (Fig. S1 a, supplementary data). The region between *δ* 3.0–5.0 ppm showed the abundance signals of sugars: glucose, fructose and sucrose (Fig. S1 b, supplementary data). In the aromatic region between *δ* 5.0–10.0 ppm (Fig. S1 c, supplementary data), alkaloids, phenols, aromatic amino acids as well as purine and pyrimidine nucleosides were identified. Alkaloid and phenolic compounds were previously reported in beans [48, 49]. Figure 3 illustrates the visible quantitative changes in the metabolites observed in the ^1^H NMR stacked spectra of the control, Se, Pb-, and SP samples. Based on the data, it is evident that the SP and Pb-samples exhibit greater variation than control compared to the Se samples. Previous studies have reported significant metabolic changes in response to Pb stress [9].

**Table 1:** List of metabolites identified in the polar extract of *Vicia faba* leaves

	**Compound name**	**Chemical formula**	^**13**^**C chemical shift (ppm)** (functional group or specific C)	^**1**^**H chemical shift (ppm)** (functional group or specific H, multiplicity of peak)	**Coupling constant** *J* (Hz)	**C**	**Pb**	**Se**	**SP**
1	1,3-Dimethylurate	C_7_H_8_N_4_O_3_	30.5 (CH_3_) 32.8 (CH_3_)	3.31 (CH_3_, s) 3.44 (CH_3_, s)	- -	√	√	√	√
2	2-Aminoadipate	C_6_H_11_NO_4_	32.8 (C^δ^) 57.4 (C^α^)	2.23 (H^δ^, t) 3.72 (H^α^, m)	7.4 -	√	√	√	√
3	2-Hydroxyisovalerate	C_5_H_10_O_3_	18.2 (CH_3_) 21.3 (CH_3_) 34.1 (CH)	0.84 (CH_3_, d) 0.95 (CH_3_, d) 2.00 (CH, m)	6.87 6.89 –	√	√	√	√
4	2-Methylglutarate	C_6_H_10_O_4_	19.9 (CH_3_) 33.4 (CH_2_) 38.4 (CH_2_) 45.3 (CH)	1.05 (CH_3_, d) 1.66 (CH_2_, m) 2.14 (CH_2_, t) 2.24 (CH, m)	6.7 - 6.8 -	X	√	X	√
5	3-Methyladipate	C_7_H_12_O_4_	21.3 (CH_3_) 35.8 (CH_2_) 47.7 (CH)	0.89 (CH_3_, d) 1.43 (CH_2_, m) 1.88 (CH, m)	6.4 - -	X	√	X	√
6	4-Aminobutyrate	C_4_H_9_NO_2_	26.4 (CH_2_) 37.0 (CH_2_) 42.0 (CH_2_)	1.90 (CH_2_, m) 2.28 (CH_2_, t) 2.99 (CH_2_, t)	- 7.3 7.4	√	X	√	X
7	Adenosine	C_10_H_13_N_5_O_4_	76.6 (CH) 155.2 (CH) 143.3 (CH)	4.79 (CH, dd) 8.24 (CH, s) 8.33 (CH, s)	8.9, 6.7 - -	√	X	√	√
8	Alanine	C_3_H_7_NO_2_	53.5 (C^α^) 18.9 (C^β^)	3.78 (H^α^, q) 1.46 (H^β^, d)	7.2, 7.2	√	√	√	√
9	AMP	C_10_H_14_N_5_O_7_P	89.5 (CH) 155.2 (CH) 142.6 (CH)	6.12 (CH, d) 8.24 (CH, s) 8.59 (CH, s)	5.6 - -	√	√	√	√
10	Arginine	C_6_H_14_N_4_O_2_	26.6 (C^γ^) 30.3 (C^β^) 57.1 (C^α^) 43.3 (C^σ^)	1.64 (H^γ^, m) 1.92 (H^β^, m) 3.75 (H^α^, t) 3.23 (H^σ^, t)	- - 6.2	√	√	√	√
11	Asparagine	C_4_H_8_N_2_O_3_	54.2 (C^α^) 37.4 (C^β^)	4.01 (H^α^, q) 2.93 (H^β^, dd) 2.88 (H^β^, dd)	4.2 4.1, 16.9 7.7, 16.9	√	√	√	√
12	Aspartate	C_4_H_7_NO_4_	54.9 (C^α^) 39.2 (C^β^)	3.88 (H^α^, dd) 2.66 (H^β^, dd)	8.80, 2.7 17.5, 8.8	√	√	√	√
13	Betaine	C_5_H_11_NO_2_	69.2 (CH_2_) 56.2 (CH_3_)	3.90 (H^α^, s) 3.25 (H^β^, s)	- -	√	√	√	√
14	Choline	C_5_H_14_NO	58.6 (C^α^) 56.8 (C^γ^) 70.4 (C^β^)	4.06 (H^α^_,_ m) 3.21 (H^γ^, s) 3.52 (H^β^, m)	- - -	√	√	√	√
15	Citrate	C_6_H_8_O_7_	48.4 (CH_2_) 48.4 (CH_2_)	2.52 (CH_2,_ d) 2.65 (CH_2,_ d)	2.5 2.6	√	√	√	√
16	Creatine	C_4_H_9_N_3_O_2_	56.4 (CH_3_) 39.5 (CH_2_)	3.01 (CH_3_, s) 3.92 (CH_2_, s)	- -	√	√	√	√
17	Dimethylamine	C_2_H_7_N	37.0 (CH_3_)	2.73 (CH_3_, s)	-	√	√	√	√
18	DL-Dopa	C_9_H_11_NO_4_	38.3 (C^β^) 58.7 (C^α^) 119.6 (C^γ^)	3.14 (H^β^_,_ q) 3.92 (H^α^_,_ t) 6.81 (H^γ^_,_ q)	6.3	√	√	√	√
19	Epinephrine	C_9_H_13_NO_3_	35.8 (CH_3_) 57.4 (CH) 71.1 (CH)	2.67 (CH_3_, s) 3.25 (CH, d) 4.93 (CH, t)	- 5.7 5.7	X	√	X	√
20	Ethanol	C_2_H_6_O	119.5 (CH_3_) 60.3 (CH_2_)	1.17 (CH_3_, t) 3.63 (CH_2_, q)	6.7 6.7	√	√	√	√
21	Formate	CH_2_O_2_	-	8.44 (CH, s)	-	√	√	√	√
22	Fructose	C_6_H_12_O_6_	78.2 (^3^CH) 66.1 (^6^CH_2_) 72.0 (^5^CH) 66.6 (^1^CH)	4.12 (^3^CH, dd) 4.02 (^6^CH_2_, dd) 4.00 (^5^CH, m) 3.56 (^1^CH, m)	12.7, 1.0 7.7, 5.4	√	√	√	√
23	Fumarate	C_4_H_4_O_4_	138.0 (CH)	6.49 (CH, s)	-	√	√	√	√
24	Gallate	C_7_H_6_O_5_	111.8 (CH)	7.00 (CH, s)	-	√	√	√	√
25	Glucose	C_6_H_12_O_6_	98.9 (^1α^CH) 94.8 (^1β^CH) 74.4 (^2α^CH) 72.6 (^4^CH) 63.7 (^6^CH)	4.66 (^1α^CH, d) 5.24 (^1β^CH, d) 3.53 (^2α^CH, m) 3.41 (^4^CH, m) 3.74 (^5β^CH, m)	7.8 3.6 - - -	√	√	√	√
26	Glutamate	C_5_H_9_NO_4_	29.7 (C^β^) 36.2 (C^γ^) 57.3 (C^α^)	2.04 (H^β^, m) 2.35 (H^γ^, m) 3.74 (H^α^, dd)	- - 7.1, 4.7	√	√	√	√
27	Glutamine	C_5_H_10_N_2_O_3_	33.7 (C^γ^) 29.1 (C^β^) 57.0 (C^α^)	2.46 (H^γ^, m) 2.14 (H^β^, m) 3.78 (H^α^, t)	- - 6.3	√	√	√	√
28	Glutarate	C_5_H_8_O_4_	26.6 (CH_2_) 40.0 (CH_2_)	1.77 (CH_2_, m) 2.17 (CH_2_, t)	- 7.4	√	X	√	X
29	Glycerate	C_3_H_6_O_4_	66.9 (CH_2_) 76.1 (CH)	3.71 (CH_2_, dd) 3.81 (CH_2_, dd)	11.4, 5.6 11.4, 5.6	√	√	√	√
30	Glycine	C_2_H_5_NO_2_	44.3 (C^α^)	3.56 (H^α^, s)	-	√	√	√	√
31	Glycolate	C_2_H_4_O_3_	64.0 (CH_2_)	3.90 (CH_2_, s)	-	√	√	√	√
32	Histidine	C_6_H_9_N_3_O_2_	30.8 (CH_2_) 57.2 (CH) 119.9 (CH)	3.17 (CH_2_, dd) 3.97 (CH, q) 7.08 (CH, s)	6.3 6.3 -	√	√	√	√
33	Isobutyrate	C_4_H_8_O_2_	22.0 (CH_3_)	1.05 (CH_3_, d) 2.37 (CH, m)	7.02 -	√	√	√	√
34	Isoleucine	C_6_H_13_NO_2_	26.8 (C^γ^) 17.5 (C^γ^) 13.9 (C^δ^)	1.29 (H^γ^, m) 1.00 (H^γ^, d) 0.91 (H^δ^, t)	- 7.0 7.1	√	√	√	√
35	Isovalerate	C_5_H_10_O_2_	26.2 (CH_3_) 51.4 (CH)	0.89 (CH_3_, d) 2.04 (CH, d)	6.6 0.5	√	√	√	√
36	Lactate	C_3_H_6_O_3_	22.17 (CH_3_)	1.33 (CH_3_, d)	6.8	√	√	√	√
37	Leucine	C_6_H_13_NO_2_	24.7 (C^δ^)	0.98 (H^δ^, t)	6.1	√	√	√	√
38	Malate	C_4_H_6_O_5_	45.2 (CH_2_) 45.4 (CH_2_)	2.36 (CH_2_, dd) 2.65 (CH_2_, dd)	15.3, 10.2 15.3, 2.9	√	√	√	√
39	Malonate	C_3_H_4_O_4_	50.2 (C^β^)	3.12 (H^β^, s)	-	√	√	√	√
40	Methionine	C_5_H_11_NO_2_S	56.8 (C^α^) 31.5 (C^γ^)	3.86 (H^α^, t) 2.63 (H^γ^, t)	6.2 6.6	√	√	√	√
41	O-Phosphocholine	C_5_H_15_NO_4_P	56.5 (CH_3_) 86.9 (CH_2_) 60.6 (CH_2_)	3.20 (CH_3_, s) 3.58 (CH_2_, m) 4.15 (CH_2_, m)	- - -	√	√	√	√
42	Phenylalanine	C_9_H_11_NO_2_	39.2 (C^β^) 58.9 (C^α^) 132.1 (C^δ^) 130.4 (C^ζ^) 31.8 (C^ε^)	3.09 (H^β^, dd) 3.99 (H^α^, dd) 7.33 (H^δ^, m) 7.36 (H^ζ^, m) 7.41 (H^ε^, m)	7.0 6.3 - - -	√	√	√	√
43	Proline	C_5_H_9_NO_2_	64.2 (C^α^) 49.0 (C^δ^) 31.9 (C^β^)	4.13 (H^α^, m) 3.41 (H^δ^, m) 2.36 (H^β^, m)	- - -	√	√	√	√
44	Pyroglutamate	C_5_H_7_NO_3_	27.9 (C^γ^) 60.9 (C^α^)	2.40 (H^γ^, m) 4.16 (H^α^, dd)	- 9.0, 5.9	√	√	√	√
45	Pyruvate	C_3_H_4_O_3_	29.3 (CH_3_)	2.35 (CH_3_, s)	-	√	√	√	√
46	Sarcosine	C_3_H_7_NO_2_	35.5 (CH_3_) 53.4 (CH_2_)	2.73 (CH_3_, s) 3.61 (CH_2_, s)	- -	√	√	√	√
47	Succinate	C_4_H_6_O_4_	37.1 (CH_2_)	2.41 (CH_2_, s)	-	√	√	√	√
48	Sucrose	C_12_H_22_O_11_	95.1 (^1^CH) 79.8 (^3’^CH) 76.8 (^4’^CH) 73.9 (^2^CH) 72.1 (^3^CH) 65.3 (^6^CH_2_) 64.2 (^1’^CH_2_) 72.1 (^3^CH)	5.42 (^1^CH, d) 4.22 (^3’^CH, d) 4.06 (^4’^CH, t) 3.56 (^2^CH, m) 3.48 (^3^CH, m) 3.83 (^6^CH_2_, m) 3.69 (^1’^CH_2_, s) 3.48 (^3^CH, m)	3.89 8.8 38.6 - - - - -	√	√	√	√
49	Threonine	C_4_H_9_NO_3_	63.4 (C^α^) 23.0 (C^γ^)	3.55 (H^α^, d) 1.33 (H^γ^, d)	5.20 6.49	√	√	√	√
50	Thymine	C_5_H_6_N_2_O_2_	13.9 (CH_3_) 142.0 (CH)	1.86 (CH_3_, s) 7.36 (CH, s)	- -	√	√	√	√
51	Trigonelline	C_7_H_7_NO_2_	148.2 (^2^CH) 147.4 (^4,6^CH) 130.5 (^5^CH) 51.1 (^1^CH_3_)	9.10 (^2^CH, s) 8.84 (^4,6^CH, t) 8.08 (^5^CH, t) 4.42 (^1^CH_3_, s)	8.80 7.37	√	√	√	√
52	Tryptophan	C_11_H_12_N_2_O_2_	121.1 (C^ζ3^) 124.8 (C ^η2^) 127.9 (C ^δ2^) 114.7(C ^ς2^)	7.18 (H^ζ3^, m) 7.27 (H^η2^, m) 7.31 (H^δ2^, s) 7.52 (H^ς2^, ddd)	- - - 7.0, 1.4, 0.5	√	√	√	√
53	Tyrosine	C_9_H_11_NO_3_	38.2 (C^β^) 58.9 (C^α^) 133.4 (C^γ^)	3.10 (H^β^, dd) 3.91 (H^α^, q) 7.18 (H^γ^, d)	7.20, 14.30	√	√	√	√
54	Tyramine	C_8_H_11_NO	118.5 (CH) 133.0 (CH)	6.89 (CH, d) 7.18 (CH, d)	7.2	√	√	√	√
55	UMP	C_9_H_13_N_2_O_9_P	105.2 (CH) 90.9 (CH) 144.7 (CH)	5.97 (CH, m) 5.98 (CH, m) 8.10 (CH, d)	- -	√	√	√	√
56	Uridine	C_9_H_12_N_2_O_6_	72.0 (CH) 92.0 (CH)	4.21 (CH, dd) 5.90 (CH, d)	9.0, 4.3 9.0	√	√	√	√
57	Valine	C_5_H_11_NO_2_	63.0 (C^α^) 20.8 (C^γ^) 19.5 (C^γ^)	3.60 (H^α^, d) 0.98 (H^γ^, d) 1.03 (H^γ^, d)	4.54 7.00 7.00	√	√	√	√
58	Xanthine	C_5_H_4_N_4_O_2_	140.3 (CH)	7.90 (CH, s)	-	√	√	√	√

**Fig. 4:**
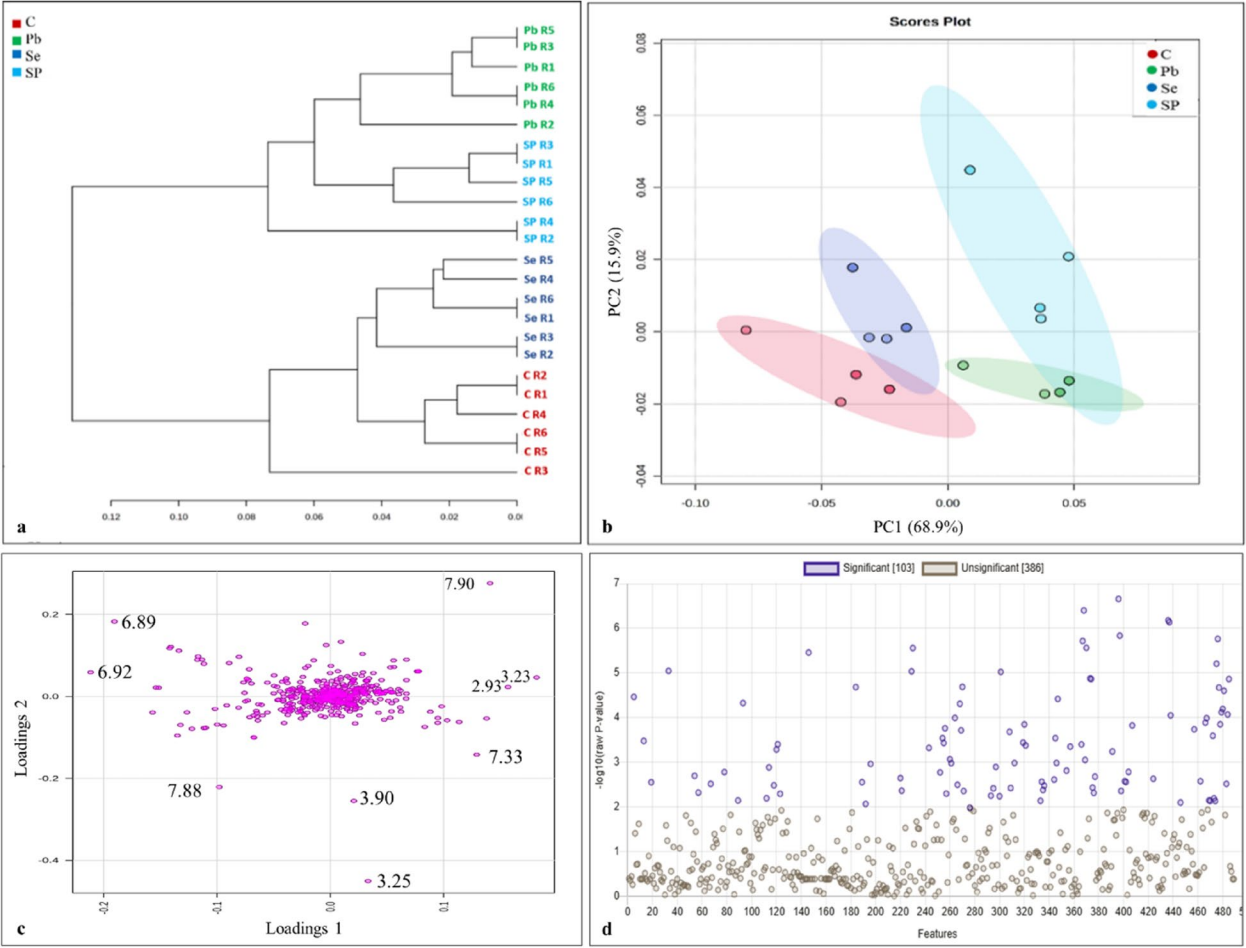
Statistical analysis of the polar extract of *Vicia faba* shoots (C = control, Pb = lead treated samples, Se = selenium treated samples, SP = samples treated with lead and selenium) of normalized data generated by MetaboAnalyst 5.0 software. **a** 2D scores plot of principal component analysis, the ovals indicate 95% Hotellings confidence intervals. PC1 and PC2 explain a total of 84.8% of the variance. **b** Hierarchical Cluster Analysis (HCA). **c**, PCA loadings plot derived from.^1^H NMR data, and **d**, One-way Analysis of Variance (ANOVA) and post-hoc tests indicate significant metabolite changes between groups at a p-value threshold of ≤ 0.05

Hierarchical cluster analysis (HCA) was utilized to comprehend the distribution of these metabolites using the metabolomic profiling generated from each treatment. The first cluster comprised the samples that were exposed to Pb and were further divided into two subclusters: one for the Pb group and the other for the SP group. The second cluster was divided into two subclusters as well: one for the Se primed plants and one for the control plants (Fig. 4a). PCA scores plot (Fig. 4b), shows the grouping of the samples based on their metabolic profiles. The first two components (PC1 and PC2) explained a significant amount of the total variance (68.9% and 15.9%, respectively). Samples from the control group are well separated from the Pb and SP samples, indicating distinct metabolic profiles. There is some overlap between the Pb and SP groups, although the groups are mostly distinct, confirming the clustering patterns observed in the HCA dendrogram. HCA and PCA indicated that the metabolic alterations induced by the addition of Pb exceed those resulting from the addition of Se in the plant. The significant impact of Pb on the metabolic profiling of plants has been documented in previous studies [50, 51].

The loadings plot (Fig. 4c) identified the variables that facilitated clustering and differentiation among the samples in the PCA scores plot. The assignment of these signals resulted in the identification of metabolites responsible for the separation on the scores plot. The loadings plot indicated that the key metabolites that contributed to the observed separation were xanthine (7.90 ppm), arginine (3.23 ppm), asparagine (2.93 ppm), phenylalanine (7.33 ppm) and tyrosine (3.90 ppm). Additionally, betaine, tyramine, and histidine, corresponding to chemical shifts at 3.25, 6.89, and 7.88 ppm respectively, also contributed significantly to the separation. Several of these metabolites have been documented to enhance plant tolerance under abiotic stress conditions. Asparagine accumulation in wheat plants under stress conditions has been documented [52]. Xanthine metabolism triggers reactive oxygen species and abscisic acid signaling in response to low temperature stress in sugar beet plants [53].

**Fig. 5:**
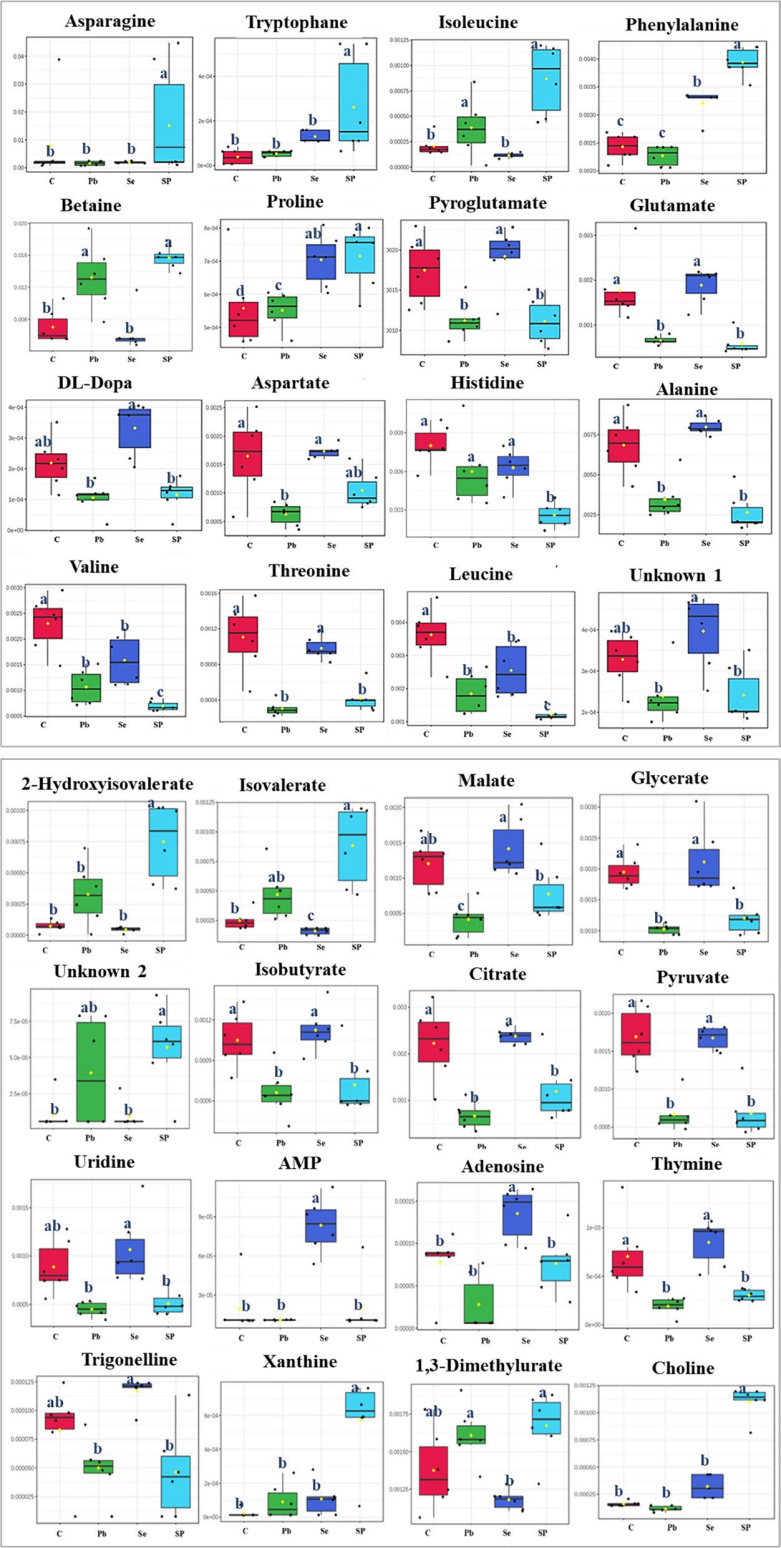
Boxplots of relative concentrations of the most significant metabolites selected from ANOVA analysis that changed between control (C), lead (Pb), selenium (Se), and lead + selenium (SP) treatments. The black dots represent the concentration of the selected metabolite in each replicate. The notch indicates the 95% confidence interval around the median of each treatment, defined as ± 1.58* IQR/sqrt (n). The mean concentration of each treatment is indicated by the yellow diamond. The Y axis defines the relative abundances of the specific metabolite and the X axis defines the treatment group. The small letters (a, b, c,…) assigned to each column indicate the significance between mean of the groups being compared at p ≤ 0.05 level according to One-Way ANOVA

The ANOVA analysis identified a total of 489 metabolites, with 103 metabolites exhibiting significant differences in concentration across the studied groups at a significance level of p ≤ 0.05 (Fig. 4d). Box plots representing the relative concentrations of the significant metabolites identified in Se-primed shoots and those exposed to Pb are shown based on ANOVA analysis at p ≤ 0.05 (Fig. 5). The corresponding p-values for these metabolites are provided in Table S1. The metabolites AMP and adenosine exhibited a significant upregulation in Se shoots compared to the other treatments. Specifically, AMP levels increased six-fold in the Se samples relative to those of the other treatments. Both AMP and adenosine serve as crucial intermediates in purine metabolism, a pathway integral to energy transfer and cellular signaling [54]. As precursors to ATP (adenosine triphosphate) and ADP (adenosine diphosphate), AMP and adenosine play vital roles in maintaining cellular energy homeostasis [55]. Previous studies have shown that AMP regulates a variety of physiological processes and accumulates in tomato plants during the ripening process [56].

Furthermore, Se samples exhibited a significant accumulation of bioactive metabolites, including trigonelline and DL-dopa. These metabolites demonstrated significantly higher concentrations in Se shoots compared to Pb and SP samples. Although the concentrations of these metabolites did not show significant differences between the Se and control treatments, they were higher in the Se shoots. For instance, trigonelline was upregulated by 1.4-fold relative to the control shoots. Trigonelline is a plant alkaloid that has been identified in other members of the family Fabaceae like *Glycine max* [57]. It is generated as an osmoregulator in response to environmental stimuli [58]. It possesses anti-cancer and antibacterial properties and plays a significant role in cell cycle regulation [59, 60]. The accumulation of trigonelline in response to Se addition has been reported in *Trigonella foenum-graecum* [61]. DL-dopa is a precursor with a broad range of clinical applications, including the production of alkaloids and catecholamines [62]. It is a precursor to the neurotransmitter’s dopamine and epinephrine, and DL-dopa levels have been found to be strongly associated with winter wheat resistance to grain aphids [63].

A significant downregulation of amino acids including aspartate and glutamate was observed in the Pb-treated samples (Pb and SP). This finding aligns with previous studies demonstrating that heavy metals, such as Pb, disrupt amino acid metabolism and key biochemical pathways, ultimately impairing plant growth [64, 65]. Aspartate, a critical amino acid, plays an essential role in plant growth and development, primarily through its interaction with phytohormones such as auxin and ethylene. It serves as a precursor for the biosynthesis of four essential amino acids: methionine, threonine, lysine, and isoleucine, and is involved in the formation of various proteins, amino acids, nucleotides, nicotinamide adenine dinucleotides (NADs), organic acids, and other metabolites [66]. Glutamate, another key metabolite, is central to nitrogen assimilation in plants. It acts as the primary amino donor in transamination reactions and is a precursor for important compounds such as glutathione and folate. The regulation of glutamate metabolism and signaling is vital for integrating plant growth and defense mechanisms [67]. The downregulation of specific amino acids in response to Pb stress has been also observed in *Solanum melongena* shoots [9].

Similarly, in this study, a significant decrease in organic acids, including citrate, malate and pyruvate, was observed in the Pb and SP samples. This finding is consistent with the results of Wang et al. [10] who reported similar reductions in citrate and malate in radish roots exposed to Pb and Cd stress. Pyruvate is a precursor in alkaloid biosynthesis via the pyruvic acid pathway as described by Ahmed et al. [68]. All terpenes are biosynthesized by the common precursor isopentyl diphosphate (IDP) and its isomer dimethyl-allyl diphosphate (DMAPP or DMADP). Both are synthesized by distinct mechanisms, namely the chloroplast pathway and the cytosolic pathway. Within the chloroplast pathway, the condensation process of pyruvate and glyceraldehyde-3-phosphate leads to the production of 2-C-methyl-D-erythritol-4-phosphate (MEP). Subsequently, MEP undergoes a conversion process to generate 4-hydroxy-3-methylbut-2-enyl diphosphate, which is further transformed into IDP and DMADP which may explain why it is downregulated in SP-stressed shoots while xanthine is increased. Uridine was also downregulated in the Pb and SP samples. It has been identified as a key factor in regulating cell division and primordia formation in pea root nodules [69]. In contrast, betaine was upregulated in both SP and Pb samples compared to the control (C) and Se samples. The upregulation of betaine in (SP); Se-primed shoots may contribute to Pb stress mitigation. Betaine plays a key role in protecting macromolecules and facilitating the detoxification of reactive oxygen species (ROS) during abiotic stress [70]. Additionally, betaine enhances the stability of thylakoid membranes and photosynthetic pigments, which contributes to the preservation of the structural integrity and optimal function of the photosynthetic system under stress conditions. [71, 72].

On the other hand, 2-methylglutarate, 3-methyladipate and epinephrine were detected in shoots exposed to Pb (Table 1). Epinephrine is a catecholamine that is utilized in the pharmaceutical industry to treat severe allergic reactions [73]. Epinephrine participates in the metabolism of glucose in plants and has been shown to regulate a variety of plant hormones [74]. The production of epinephrine has been shown to be enhanced in plants under stress conditions [75]. Our findings reveal that a drop in DL-dopa levels in Pb samples is accompanied by the detection of epinephrine, implying that DL-dopa to epinephrine conversion occurs in these samples.

Metabolite profiling of Se-primed plants exposed to Pb (SP shoots) revealed significant alterations compared to other treatments, suggesting that Se priming induces a stress memory effect that enhances resilience to subsequent stress, as evidenced by improved vegetative growth parameters comparing to Pb samples (Fig. 1). Among the metabolites, asparagine, tryptophan, xanthine, phenylalanine, and choline were significantly upregulated in SP shoots relative to other treatments, indicating their roles in enhancing stress tolerance. Asparagine, a key nitrogen storage and transport molecule, is crucial for plant metabolism and plays an important role in mitigating heavy metal stress. It has been shown to form complexes with heavy metals, reducing their toxicity. For instance, asparagine has been reported to bind with zinc in *Deschampsia cespitosa*, mitigating zinc toxicity [75]. The upregulation of asparagine in SP plants, but not in Pb plants, suggests that Se priming may enhance the plant’s ability to utilize asparagine in response to Pb stress. This aligns with findings indicating that asparagine accumulates in plants exposed to Pb and other heavy metals, acting as a protective agent against stress [76, 77]. Additionally, asparagine functions as an osmolyte in wheat seedlings during abiotic stress conditions [78]. Tryptophan plays a protective role against heavy metal stress, particularly in the case of Pb and nickel, as it has the ability to chelate these metals and mitigate their harmful effects [77]. Exogenous application of tryptophan has been shown to enhance heavy metal stress tolerance in *Brassica oleracea* and *Helianthus annuus* plants [79, 80]. Tryptophan is also a precursor for auxin (IAA), a plant hormone that regulates growth and stress responses. Auxins play a critical role in stress adaptation by reducing metal uptake, promoting metal chelation, and mitigating oxidative damage under stressful conditions [81]. It has been reported that stress conditions trigger alterations in auxin production and signaling pathways, facilitating cellular reprogramming that supports plant growth and development under stress [82].

Additionally, the upregulation of phenylalanine and choline in SP shoots could contribute to the production of osmo-protectants and secondary metabolites, which are crucial for plant growth and defense against Pb stress. Phenylalanine serves as a precursor for several secondary metabolites, such as polyphenols, which are vital for seedling growth, development, and stress tolerance in plants [83]. Choline, a precursor of glycine betaine, an important osmo-protectant metabolite, was also upregulated in SP-treated plants. Foliar application of choline has been shown to alleviate the negative effects of cadmium toxicity by reducing cadmium uptake in *Solanum lycopersicum* seedlings [84].

Proline metabolites was upregulated in both Se and SP samples. Seed priming with selenium has been reported to induce the accumulation of different metabolites that improve plant growth and mitigate abiotic stress conditions like drought, flooding, and heavy metal stress [85–87]. For example, Se has been reported to enhance proline accumulation, which serves as an osmotic regulator and protects plants under drought stress [87].

### Biological pathways

**Fig. 6:**
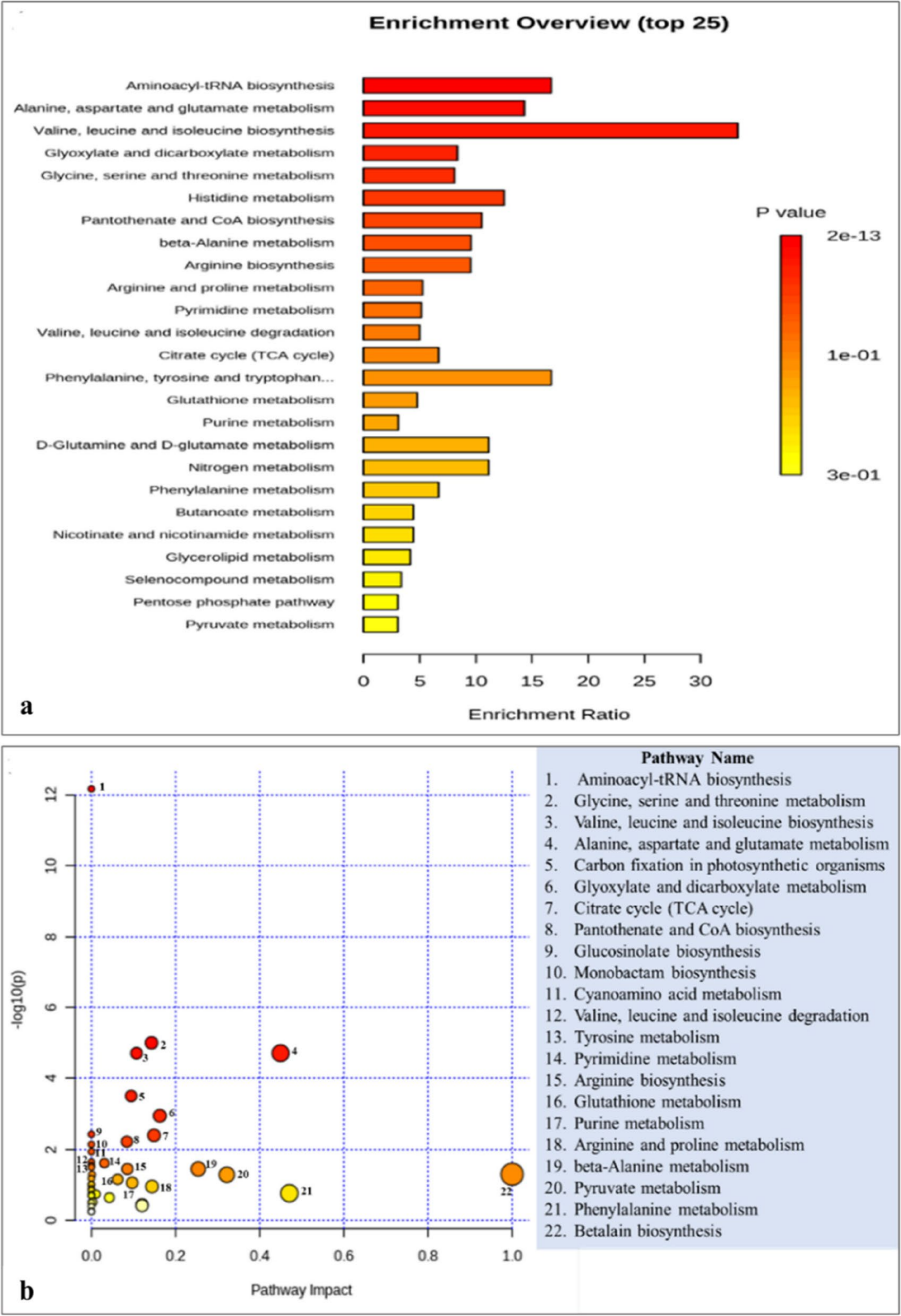
Metabolic pathways in *Vicia faba* polar extract that are significantly altered in Se-primed shoots and/or foliar sprayed with Pb. **a** Interactive bar-chart of the enrichment analysis (based on KEGG database). The most significant *p*-values are represented by dark red columns, while the color decreases gradually with decreasing *p*-values, with pale yellow representing the least significant; the length of the column represents the enrichment ratio. **b** Pathway analysis (based on KEGG database) showing significantly changed metabolic pathways in response to Se and/or Pb treatments. The dark red circles indicate the pathways that were strongly affected by stress; As the *p*-value increases, the color progressively fades, whereas the larger the circle size, the greater the pathway's influence

**Fig. 7:**
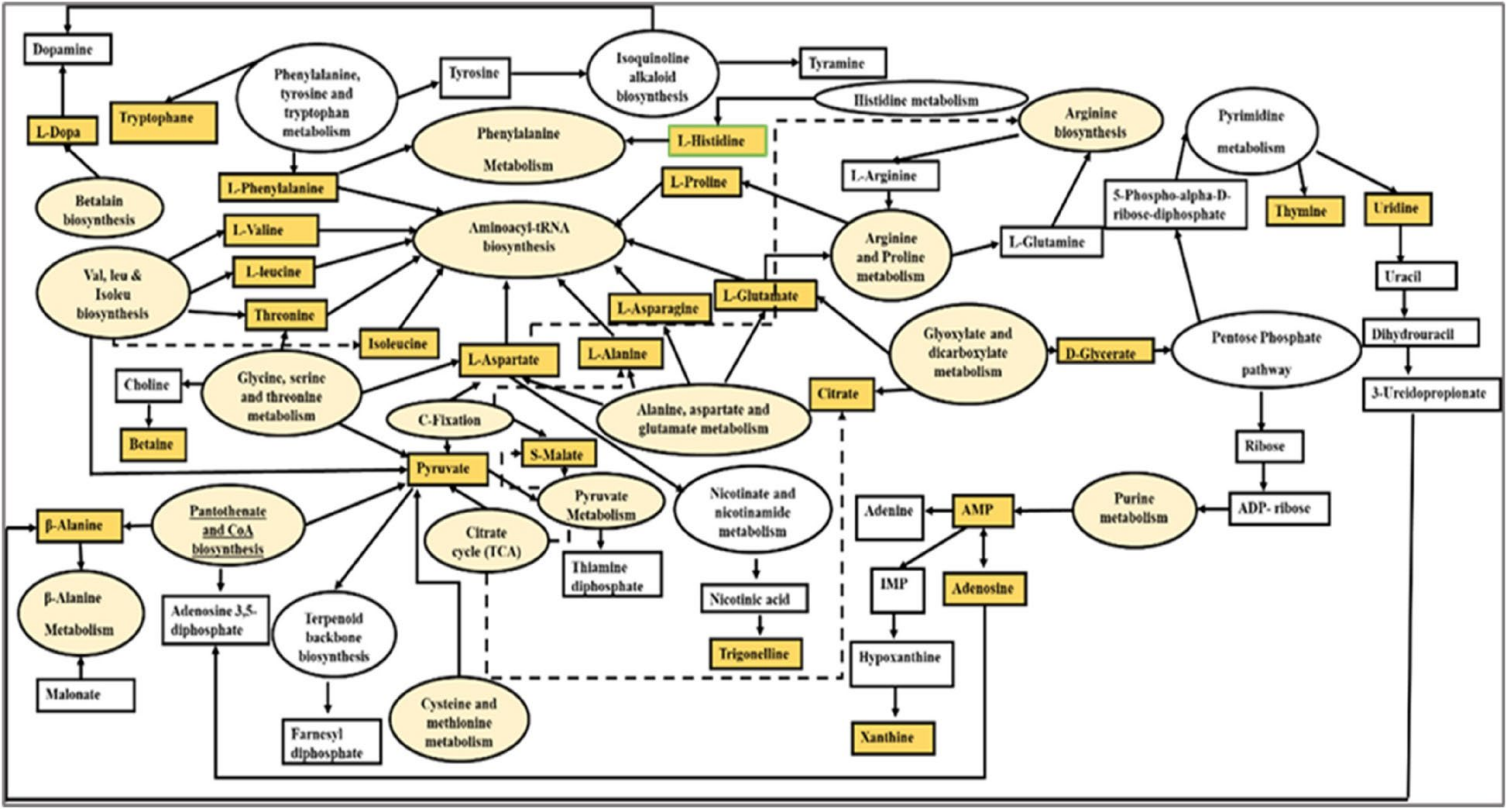
MetaboAnalyst 6.0 and the KEGG database were utilized to explain various metabolic pathways that were significantly altered by the addition of Se and/or Pb (marked in yellow ovals), resulting in catabolism and anabolism of the significant metabolites (marked in orange boxes)

The addition of Se and Pb significantly altered 42 metabolic pathways, as revealed by pathway analysis (supplementary Table S2). The most substantially altered pathways were aminoacyl-tRNA biosynthesis; alanine, aspartate, and glutamate metabolism; and valine, leucine, and isoleucine biosynthesis, as determined by both enrichment analysis and pathway analysis (Fig. 6a). The betalain biosynthesis pathway represented the primary impacted pathway based on pathway analysis (Fig. 6b). In the aminoacyl-tRNA biosynthetic pathway, ten significant metabolites were implicated, such as proline, arginine, isoleucine, and glutamine (Fig. 7). The physiological responses of plants to adverse conditions are significantly influenced by aminoacyl-tRNA biosynthesis. The research conducted by Fu et al. [88], and Baranašić et al. [89] demonstrated that aminoacyl-tRNA biosynthesis is an essential pathway for the synthesis of proteins that play a significant role in the stress response. Additionally, tRNAs have the ability to regulate the level of aminoacylation in response to stress. The aminoacyl-tRNA production pathway of *Elodea nuttallii* plants was significantly altered when exposed to mercury and methylmercury [90]. Also, *Solanum nigrum* plants that were exposed to heavy metals Cd and Zn showed a substantial change in this pathway [91]. Additionally, this pathway was identified as the most significantly affected when the *Oryza sativa* plants were exposed to nano TiO_2_ and Cd [92]. In the present investigation, glutamate and histidine are critical intermediates in the biosynthesis of amino acid-tRNA that were downregulated in plants that were exposed to Pb. This suggests that Pb disrupts the translation pathway by disrupting the tRNA recognizing codons and transporting amino acids, resulting in a decrease in the total protein content [9]. It is suggested that the Se-primed plants undergo an opposite process, regardless of whether they are exposed to Pb. This results in an increase in the overall protein content, which in turn increases the measured growth attributes. The alanine, aspartate, and glutamate metabolism pathway in bean shoots was substantially altered in the present study as a result of Se priming and Pb stress. Glutamate and aspartate are amino acids that are essential for a plant's response to heavy metal stress. They are involved in a variety of metabolic processes that help plants combat the detrimental effects of heavy metals. For instance, glutamate functions as a precursor for proline and glutathione [93, 94]. Glutathione has the potential to safeguard plant cells from the detrimental effects of heavy metals through three potential mechanisms: first, it directly neutralizes ROS; second, it functions as a metal chelator, preventing heavy metals from interfering with essential cellular components; and third, it acts as a precursor to the production of phyto- chelating metabolites [93]. Proline was proved to assist plants in maintaining turgor pressure and cell integrity during stress conditions [95]. It was upregulated in Se-primed groups and downregulated in Pb-stressed plants, indicating that the proline metabolism pathway was altered in Pb-stressed plants. Similar to this, Liu and his colleagues [29] have recently reported that the expression of the PK, GPT, P5CS, SUS, SPS, and CYP98A genes was increased when apple roots were treated with Se. Furthermore, a substantial quantity of osmo-regulating substances, such as citric acid, L-proline, D-sucrose, and chlorogenic acid were accumulated. Aspartate is an essential amino acid in the context of plant growth and development, as it is involved in a variety of metabolic pathways, such as glycolysis and the tricarboxylic acid (TCA) cycle. Furthermore, it is integrated into the process of protein synthesis and provides a protective function for plants in the presence of heavy metal stress [96]. Also, aspartate participates in the biosynthesis of glycine betaine [66]. The valine, leucine, and isoleucine biosynthesis pathway is responsible for the biosynthesis of essential, branched-chain amino acids. These amino acids are utilized as compounds in other biological pathways, such as the aminoacyl t-RNA biosynthesis pathway, and therefore play a critical role in plant growth and development [97]. As observed in seedlings that have been exposed to Pb, the catabolism of these amino acids contributes to a variety of physiological processes in plants [98]. Research has demonstrated that the biosynthesis pathway for valine, leucine, and isoleucine is substantially altered in the leaves of *Vicia sativa* plants that have been treated with herbicides [99].

## Conclusion

The results of this study indicate that the utilization of selenium through seed priming resulted in substantial enhancements in numerous vegetative parameters when plants were subsequently exposed to Pb stress. For example, the shoot and root lengths and weights of *Vicia faba* were significantly increased. The observed enhancement can be attributed to the facilitation of the production of essential metabolites, including amino acids, organic acids, phenols, and carbohydrates, which are essential for the growth, development, and resistance to environmental stressors of plants. Asparagine, phenylalanine, proline, tryptophan, and xanthine were all upregulated in Se-primed shoots that were exposed to Pb. It has been determined that the metabolites mentioned above can perform protective functions in the presence of stress conditions, thereby enhancing stress resilience and preserving cellular viability. Pathway analysis indicated that Se and Pb were involved in the modification of 42 pathways. The most significantly affected pathways were alanine, aspartate, and glutamate metabolism; aminoacyl-tRNA biosynthesis; and valine, leucine, and isoleucine biosynthesis. A dose–response relationship with varying concentrations of Se and Pb would be recommended for further study to prove optimal Se priming doses and ascertain the threshold of Pb tolerance.

## Supplementary Information

Supplementary Material 1.

## Data Availability

All data generated are included in the manuscript or attached as supplementary files. All data generated are included in the manuscript or attached as supplementary files.

## Abbreviations

MEP: 2-C-methyl-D-erythritol-4-phosphate
GC–MS: Gas chromatography–mass spectrometry
Pb: Lead
NADs: Nicotinamide adenine dinucleotides
N_2_: Nitrogen
ROS: Reactive oxygen species
Se: Selenium
TCA: Tricarboxylic acid
