# Supplementary material for: Metabolomic evaluation of selenium seed priming on mitigating lead stress toxicity in Vicia faba plants
Source: BMC Plant Biol. 2025 Apr 17;25:491. doi: 10.1186/s12870-025-06453-6 (PMC12004563; doi:10.1186/s12870-025-06453-6)
Supplement: Supplementary file 1 — Supplementary Material 1. [file 12870_2025_6453_MOESM1_ESM.docx]

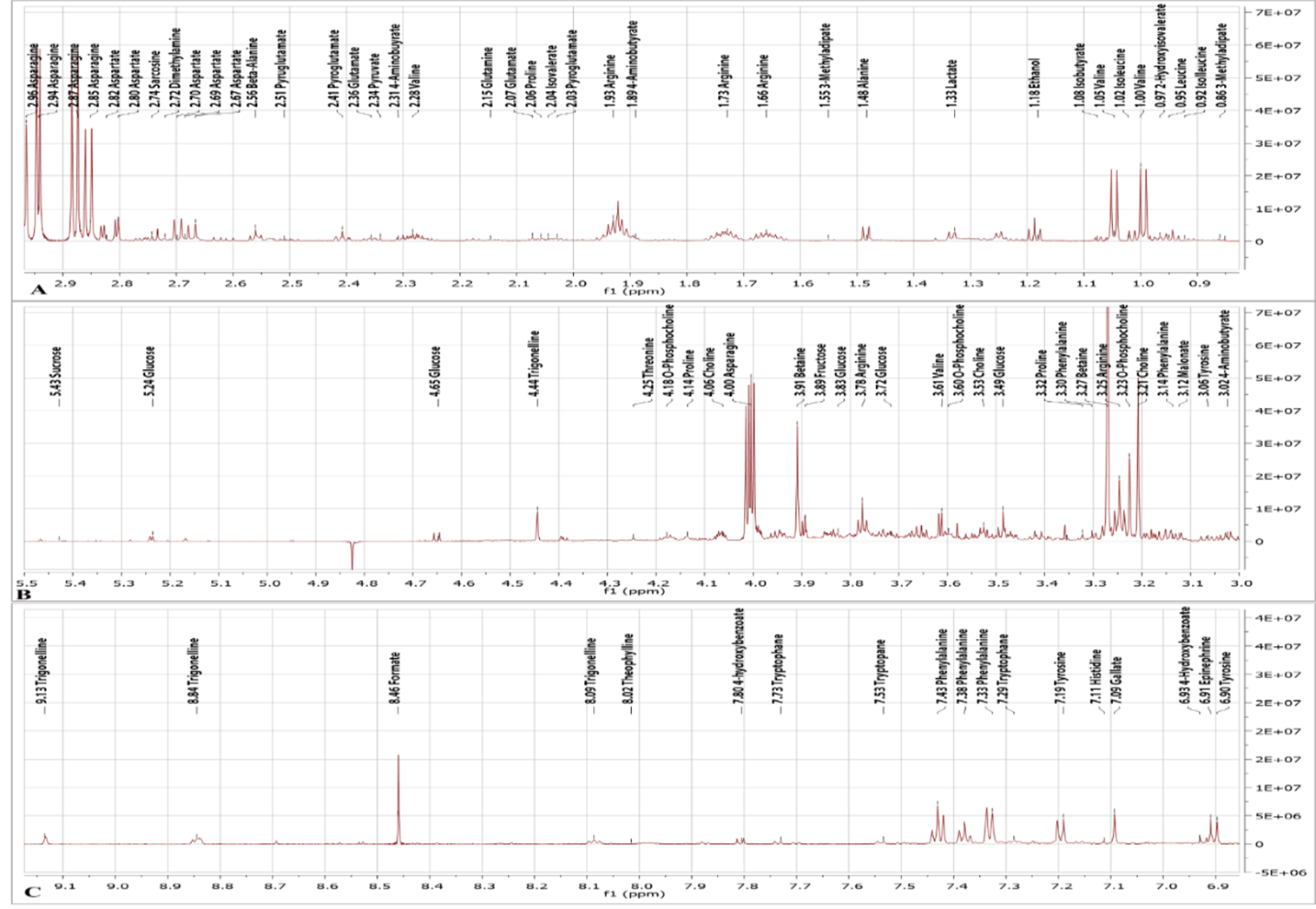


**Fig. S1** ^1^H NMR spectra of polar extract of *Vicia faba* shoots showing A) aliphatic region, B) sugar region and C) aromatic region.

**Table S1** One-way ANOVA & post-hoc Tests for the most significantly changed metabolites after Se and/or Pb addition

|  | **f.value** | **p.value** | **FDR** | **Tukey's HSD** |
| --- | --- | --- | --- | --- |
| Pyruvate | 26.174 | 3.97E-07 | 8.88E-05 | Pb-C; SP-C; Se-Pb; SP-Se |
| Alanine | 24.227 | 7.26E-07 | 8.88E-05 | Pb-C; SP-C; Se-Pb; SP-Se |
| Leucine | 21.645 | 1.72E-06 | 0.00013441 | Pb-C; Se-C; SP-C; SP-Se |
| Threonine | 20.314 | 2.76E-06 | 0.00015016 | Pb-C; SP-C; Se-Pb; SP-Se |
| AMP | 19.685 | 3.49E-06 | 0.00017053 | Se-C; Se-Pb; SP-Se |
| Valine | 18.185 | 6.21E-06 | 0.00027604 | Pb-C; Se-C; SP-C; SP-Se |
| Xanthine | 17.266 | 8.99E-06 | 0.00033094 | SP-C; SP-Pb; SP-Se |
| Betaine | 17.139 | 9.47E-06 | 0.00033094 | Pb-C; SP-C; Se-Pb; SP-Se |
| Glutamate | 16.33 | 1.33E-05 | 0.00039259 | Pb-C; SP-C; Se-Pb; SP-Se |
| Isoleucine | 16.271 | 1.36E-05 | 0.00039259 | SP-C; SP-Pb; SP-Se |
| Isovalerate | 14.899 | 2.50E-05 | 0.00058219 | SP-C; SP-Pb; SP-Se |
| Citrate | 13.996 | 3.80E-05 | 0.00080819 | Pb-C; SP-C; Se-Pb; SP-Se |
| DL-Dopa | 13.553 | 4.70E-05 | 0.00095505 | Se-Pb; SP-Se |
| 2-Hydroxyisovalerate | 12.927 | 6.39E-05 | 0.0012023 | SP-C; SP-Pb; SP-Se |
| Tryptophane | 12.364 | 8.50E-05 | 0.0014844 | SP-C; SP-Pb; SP-Se |
| Choline | 11.389 | 0.000142 | 0.0020062 | SP-C; SP-Pb; SP-Se |
| Asparagine | 11.368 | 0.000144 | 0.0020062 | SP-C; SP-Pb; SP-Se |
| Thymine | 11.292 | 0.00015 | 0.002033 | Pb-C; SP-C; Se-Pb; SP-Se |
| Glycine | 10.831 | 0.000193 | 0.0024174 | Pb-C; Se-Pb; SP-Se |
| Aspartate | 10.128 | 0.000287 | 0.0032726 | Pb-C; Se-Pb; SP-Se |
| Adenosine | 9.8739 | 0.000333 | 0.0037019 | Se-C; Se-Pb; SP-Se |
| Histidine | 9.7301 | 0.000363 | 0.0039312 | SP-C; SP-Pb; SP-Se |
| Pyroglutamate | 9.5743 | 0.000398 | 0.004052 | Pb-C; SP-C; Se-Pb; SP-Se |
| Malate | 9.3821 | 0.000446 | 0.0043661 | Pb-C; Se-Pb; SP-Se |
| Isobutyrate | 9.2763 | 0.000476 | 0.0045638 | Pb-C; Se-Pb; SP-Se |
| Phenylalanine | 9.1373 | 0.000518 | 0.0048724 | SP-C; SP-Pb; SP-Se |
| Uridine | 8.0245 | 0.00105 | 0.0088492 | SP-C; Se-Pb; SP-Se |
| Unknown 2 | 7.0622 | 0.002015 | 0.014928 | SP-C; Se-Pb; SP-Se |
| Trigonelline | 6.597 | 0.002804 | 0.018042 | Se-Pb; SP-Se |
| 1,3-Dimethylurate | 6.4832 | 0.003045 | 0.019093 | Se-Pb; SP-Se |
| Unknown 1 | 6.3795 | 0.003285 | 0.020079 | Se-Pb; SP-Se |
| Proline | 5.8046 | 0.00505 | 0.02704 | Se-C; SP-C |

**Table S2** Pathway analysis showing significantly altered metabolic pathways in *Vicia faba* polar extract. The table shows the detailed results from the pathway analysis. Since we are testing many pathways at the same time, the statistical p values from enrichment analysis are further adjusted for multiple testings. In particular, the **Total** is the total number of compounds in the pathway; the **Hits** is the matched number from the user uploaded data; the **Raw p** is the original p value calculated from the enrichment analysis; the Holm p is the p value adjusted by Holm-Bonferroni method; the **FDR p** is the p value adjusted using False Discovery Rate; the **Impact** is the pathway impact value calculated from pathway topology analysis.

|  | **Pathways** | **Total** | **Expected** | **Hits** | **Raw p** | **Log (P)** | **Holm adjust** | **FDR** | **Impact** |
| --- | --- | --- | --- | --- | --- | --- | --- | --- | --- |
| 1 | Aminoacyl-tRNA biosynthesis | 46 | 0.77546 | 12 | 6.76E-13 | 12.17 | 6.49E-11 | 6.49E-11 | 0 |
| 2 | Glycine, serine and threonine metabolism | 33 | 0.5563 | 6 | 9.93E-06 | 5.0031 | 0.00094315 | 0.000466 | 0.14292 |
| 3 | Valine, leucine and isoleucine biosynthesis | 22 | 0.37087 | 5 | 1.94E-05 | 4.7116 | 0.001826 | 0.000466 | 0.10727 |
| 4 | Alanine, aspartate and glutamate metabolism | 22 | 0.37087 | 5 | 1.94E-05 | 4.7116 | 0.001826 | 0.000466 | 0.44964 |
| 5 | Carbon fixation in photosynthetic organisms | 21 | 0.35401 | 4 | 0.000311 | 3.5078 | 0.028573 | 0.005963 | 0.09453 |
| 6 | Glyoxylate and dicarboxylate metabolism | 29 | 0.48887 | 4 | 0.001125 | 2.949 | 0.10235 | 0.017995 | 0.16264 |
| 7 | Glucosinolate biosynthesis | 65 | 1.0958 | 5 | 0.003726 | 2.4288 | 0.3353 | 0.047951 | 0 |
| 8 | Citrate cycle (TCA cycle) | 20 | 0.33715 | 3 | 0.003996 | 2.3984 | 0.35564 | 0.047951 | 0.14879 |
| 9 | Pantothenate and CoA biosynthesis | 23 | 0.38773 | 3 | 0.006003 | 2.2216 | 0.52829 | 0.064035 | 0.08423 |
| 10 | Monobactam biosynthesis | 8 | 0.13486 | 2 | 0.007182 | 2.1437 | 0.62485 | 0.068949 | 0 |
| 11 | Cyanoamino acid metabolism | 29 | 0.48887 | 3 | 0.011585 | 1.9361 | 0.9963 | 0.1011 | 0 |
| 12 | Valine, leucine and isoleucine degradation | 37 | 0.62374 | 3 | 0.022537 | 1.6471 | 1 | 0.1787 | 0 |
| 13 | Pyrimidine metabolism | 38 | 0.64059 | 3 | 0.024198 | 1.6162 | 1 | 0.1787 | 0.03089 |
| 14 | Tyrosine metabolism | 16 | 0.26972 | 2 | 0.02834 | 1.5476 | 1 | 0.19433 | 0 |
| 15 | Butanoate metabolism | 17 | 0.28658 | 2 | 0.031791 | 1.4977 | 1 | 0.1999 | 0 |
| 16 | Arginine biosynthesis | 18 | 0.30344 | 2 | 0.035399 | 1.451 | 1 | 0.1999 | 0.08544 |
| 17 | beta-Alanine metabolism | 18 | 0.30344 | 2 | 0.035399 | 1.451 | 1 | 0.1999 | 0.25397 |
| 18 | Betalain biosynthesis | 3 | 0.050573 | 1 | 0.049758 | 1.3031 | 1 | 0.24623 | 1 |
| 19 | Phenylalanine, tyrosine and tryptophan biosynthesis | 22 | 0.37087 | 2 | 0.051298 | 1.2899 | 1 | 0.24623 | 0.0015 |
| 20 | Pyruvate metabolism | 22 | 0.37087 | 2 | 0.051298 | 1.2899 | 1 | 0.24623 | 0.32193 |
| 21 | Indole alkaloid biosynthesis | 4 | 0.067431 | 1 | 0.06581 | 1.1817 | 1 | 0.30084 | 0 |
| 22 | Glutathione metabolism | 26 | 0.4383 | 2 | 0.069283 | 1.1594 | 1 | 0.30233 | 0.06248 |
| 23 | Purine metabolism | 63 | 1.062 | 3 | 0.086404 | 1.0635 | 1 | 0.36064 | 0.09692 |
| 24 | C5-Branched dibasic acid metabolism | 6 | 0.10115 | 1 | 0.097135 | 1.0126 | 1 | 0.373 | 0 |
| 25 | Isoquinoline alkaloid biosynthesis | 6 | 0.10115 | 1 | 0.097135 | 1.0126 | 1 | 0.373 | 0 |
| 26 | Arginine and proline metabolism | 34 | 0.57316 | 2 | 0.11026 | 0.95758 | 1 | 0.40712 | 0.14404 |
| 27 | Tropane, piperidine and pyridine alkaloid biosynthesis | 8 | 0.13486 | 1 | 0.12745 | 0.89466 | 1 | 0.45316 | 0 |
| 28 | Lysine biosynthesis | 9 | 0.15172 | 1 | 0.14224 | 0.84698 | 1 | 0.48768 | 0 |
| 29 | Phenylalanine metabolism | 11 | 0.18543 | 1 | 0.1711 | 0.76675 | 1 | 0.5664 | 0.47059 |
| 30 | Cysteine and methionine metabolism | 46 | 0.77546 | 2 | 0.1803 | 0.74399 | 1 | 0.57345 | 0.01139 |
| 31 | Nitrogen metabolism | 12 | 0.20229 | 1 | 0.18518 | 0.73241 | 1 | 0.57345 | 0 |
| 32 | Selenocompound metabolism | 13 | 0.21915 | 1 | 0.19903 | 0.70109 | 1 | 0.57898 | 0 |
| 33 | Nicotinate and nicotinamide metabolism | 13 | 0.21915 | 1 | 0.19903 | 0.70109 | 1 | 0.57898 | 0 |
| 34 | Histidine metabolism | 15 | 0.25287 | 1 | 0.22605 | 0.6458 | 1 | 0.63825 | 0.04264 |
| 35 | Zeatin biosynthesis | 21 | 0.35401 | 1 | 0.30196 | 0.52005 | 1 | 0.80523 | 0 |
| 36 | Glycerolipid metabolism | 21 | 0.35401 | 1 | 0.30196 | 0.52005 | 1 | 0.80523 | 0.00426 |
| 37 | Thiamine metabolism | 22 | 0.37087 | 1 | 0.3139 | 0.50321 | 1 | 0.81444 | 0 |
| 38 | Glycolysis / Gluconeogenesis | 26 | 0.4383 | 1 | 0.35971 | 0.44404 | 1 | 0.90875 | 0.12036 |
| 39 | Tryptophan metabolism | 28 | 0.47202 | 1 | 0.3815 | 0.4185 | 1 | 0.93909 | 0.12037 |
| 40 | Terpenoid backbone biosynthesis | 30 | 0.50573 | 1 | 0.40258 | 0.39514 | 1 | 0.9662 | 0 |
| 41 | Phenylpropanoid biosynthesis | 46 | 0.77546 | 1 | 0.5481 | 0.26114 | 1 | 1 | 0 |
| 42 | Porphyrin and chlorophyll metabolism | 48 | 0.80917 | 1 | 0.56369 | 0.24896 | 1 | 1 | 0 |
